# Splenogonadal Fusion Diagnosed by Doppler Ultrasonography

**DOI:** 10.1100/tsw.2004.73

**Published:** 2004-06-07

**Authors:** Jose Murillo B. Netto, Luis M. Pérez, David R. Kelly, David B. Joseph, Stuart A. Royal

**Affiliations:** Pediatric Urology, Division of Urology, Department of Surgery; Pediatric Radiology, Children's Hospital, University of Alabama at Birmingha, USA

## Abstract

Splenogonadal fusion usually presents as a left scrotal mass but rarely is the diagnosis suspected preoperatively. Herein, we present the first report of a left splenogonadal fusion which was suspected preoperatively by doppler ultrasonography in a 2 year old boy.

## INTRODUCTION

There have been approximately 145 case reports of splenogonadal fusion in the literature. In most the diagnosis was made during an operative procedure or at autopsy. In only 4 reported cases was the diagnosis suspected prior to surgery, including 2 confirmed by radionuclide scanning.7,8,9 We report a case and suggest that doppler ultrasonography may be utilized as the method of choice to diagnose splenogonadal fusion.

## CASE REPORT

A 2-year-old boy was referred with a history of a left hydrocele and scrotal mass. He had previously undergone a left hydrocelectomy at 6 months of age. At 10 months of age he was explored for a recurrent left hydrocele via a groin incision. No communication was noted at the internal ring. His mother reported an enlargement of the left hemiscrotum after the second repair. There was no evidence of hemolytic anemia or viral infection at the time he was referred to us.

On physical examination his abdomen was normal without evidence of splenomegaly. His genitalia revealed a normal phallus with a small left hydrocele and a large, firm left testis. His right testis was normal. There were no other abnormalities. Urinalysis and serum beta-hCG, AFP and LDH were normal.

An ultrasound revealed the right testis measured 0.8 x 1.4 x 1.0 cm, and the left testis measured 1.4 x 2.6 x 1.4 cm. There was a small amount of fluid around the left testis and no obvious tumor. The epididymis was not enlarged and there was no calcification within the testis. Doppler ultrasonography was not performed.

A working diagnosis of idiopathic enlargement of the left testis secondary to operative inflammation was made and conservative follow-up was recommended. Reexamination at 4 weeks and at 6 months revealed no changes. At the 6-month follow-up an unsuccessful attempt was made to aspirate the hydrocele surrounding the left scrotal mass.

At this time a doppler ultrasound of the scrotum revealed a medium level echogenic mass containing a branching arterial structure within the left hemiscrotum measuring 3.7 x 1.3 cm (fig 1 a & b). The mass had no cysts, calcifications or irregular echogenicity. There was prominent flow involving the entire mass noted with power doppler. Spectral doppler showed two components of vascular supply, one with a lower peak systolic amplitude and one with higher peak systolic amplitude. No significant venous flow was detected within the mass. The right testis had only a small amount of pulsatile flow using the same technique. There was a structure in the caudal left hemiscrotum measuring 1.0 x 0.6 cm that had the echotexture and similar flow characteristics of the right testis.

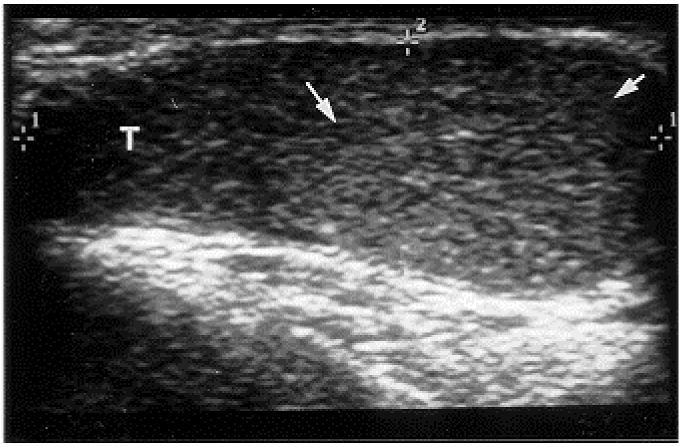

**FIGURE 1A.** Longitudinal ultrasonogram demonstrates the splenic mass (arrows) which is slightly hypoechoic positioned above the normal appearing testis (T).

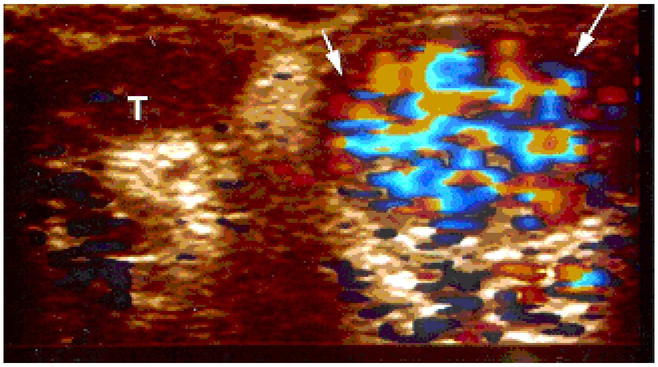

**FIGURE 1B**. Transverse Power Doppler Ultrasound of both testis demonstrates the diffuse increased flow to the splenic tissue (arrows) when compared to the small amount of normal flow seen in the right testicle (T).

The child underwent a left inguinal exploration with delivery of his testis. A large mass fused to the superior portion of the testis was found. The mass and the testis could not be separated and a radical orchiectomy was performed. Pathologic gross and microscopic examination of the specimen was consistent with the discontinuous type of splenogonadal fusion (fig 1 c & d).

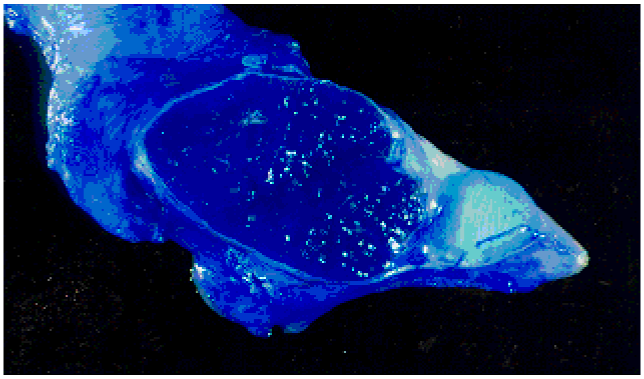

**FIGURE 1C**. Splenogonadal fusion, discontinuous type. The larger splenic mass (2.4 x 1.4 cm) lies superior to the smaller nubbin of testicular tissue (0.8 x 0.7).

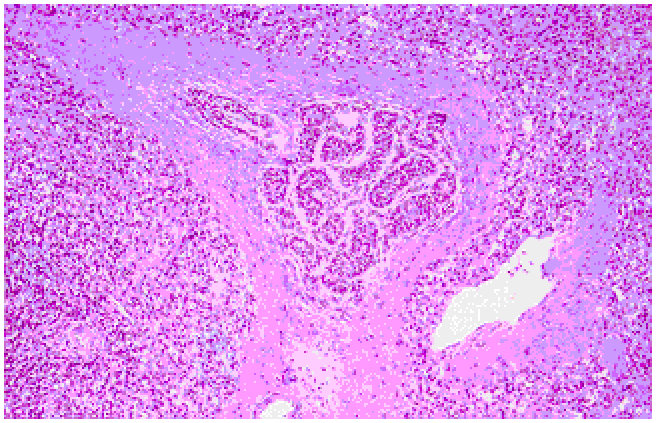

**FIGURE 1D**. An uncommon finding in splenogonadal fusion is the presence of a testicular inclusion composed of seminiferous tubules within the splenic mass.(H&E,x160).

## DISCUSSION

Splenogonadal fusion is a rare congenital anomaly whereby the left gonad is typically fused to a segment of splenic tissue. A single case of splenogonadal fusion occurring on the right side has been reported. 10 The first reports of splenogonadal fusion were by Boestroen in 18831 and detailed by Pommer in 1889.2 In 1956 Putschar and Manion3 proposed a classification in which they grouped splenogonadal fusion into 2 types. In the *continuous type* there is a cord-like structure connecting the orthotopic intraperitoneal spleen to the gonadal mesonephric structure. In the *discontinuous type* the accessory and orthotopic spleens are not connected.

There have been at least 145 cases of splenogonadal fusion reported in the literature. The largest review was made by Carragher et al.4 in 1990, describing 123 cases. A male to female ratio of approximately 16:1 was noted. The continuous type comprised 55%. The diagnosis is most often made in the first two decades of life (72% to 82%)4,5 with 50 percent of the cases diagnosed prior to 10 years of age.4,5

The clinical presentation of splenogonadal fusion is usually a scrotal mass (40%).6 Other common clinical forms of presentation include a left inguinal hernia, hydrocele, and undescended testis. Twenty to 23 % of cases are found at autopsy.4, 5 Congenital anomalies are identified in a third of cases. The majority of anomalies is associated with the continuous type of splenogonadal fusion, and includes limb defects, micrognathia and skull anomalies. 4,5

Karaman and Gonzales noted that an unsuspected diagnosis during operative exploration may have lead to an unnecessary orchiectomy in one third of the reported cases.5 In only 4 previously reported cases was the diagnosis suspected preoperatively, including 2 cases confirmed by radionuclide scanning, using radionuclide sulfur colloid imaging to demonstrate splenic uptake by the mass.7, 8, 9 In our case, orchiectomy was necessary because the splenic tissue was adherent to the testis making it difficult to separate (fig 1d).

Given that the most common clinical presentation of splenogonadal fusion is a scrotal mass, ultrasonography will likely be the first imaging study to evaluate this condition. The literature review revealed few cases where an ultrasound examination has been performed for splenogonadal fusion. However, it has been stated that a vascularized cord-like structure connecting the spleen to the splenogonadal mass11 should be investigated for by the ultrasonographer. The splenogonadal fusion mass has been mentioned to be highly vascular12, which would support the doppler finding in our case.

The differential diagnosis of the intra-scrotal paratesticular mass in children is broad, but includes malignant tumor, adrenal rest tissue, epidydimitis, orchitis, hemangioma, and hematoma.13 Testicular tumors are typically irregular in echotexture and vascularity and the majority are intratesticular. The extent of vascularity observed by doppler ultrasonography of splenogonadal fusion is more extensive than any typical paratesticular or testicular tumor and may easily be compared to the vascularity of the patients own intraabdominal spleen.

We believe that a careful examination of a left scrotal mass with doppler ultrasonography is likely to differentiate splenogonadal fusion from a tumor. A doppler ultrasound finding of an echogenic hypervascular lesion situated on the superior aspect of the testis may lead to the clinical impression of a splenogonadal fusion.
